# Eosinophilic Esophagitis: A Review of Histopathological and Diagnostic Aspects

**DOI:** 10.3390/diagnostics15233011

**Published:** 2025-11-26

**Authors:** Serena Salzano, Rosario Caltabiano, Maurizio Zizzo, Massimiliano Fabozzi, Andrea Palicelli, Magda Zanelli, Nektarios Koufopoulos, Graziano Troina, Santi Mangiafico, Giuseppe Broggi

**Affiliations:** 1 Department of Medical and Surgical Sciences and Advanced Technologies “G.F. Ingrassia” Anatomic Pathology, University of Catania, 95123 Catania, Italy; sere.salzano@gmail.com (S.S.); rosario.caltabiano@unict.it (R.C.); giuseppe.broggi@phd.unict.it (G.B.); 2 Surgical Oncology Unit, Azienda USL-IRCCS di Reggio Emilia, 42122 Reggio Emilia, Italy; massimiliano.fabozzi@ausl.re.it; 3 Pathology Unit, Azienda USL-IRCCS di Reggio Emilia, 42123 Reggio Emilia, Italy; andrea.palicelli@ausl.re.it (A.P.); magda.zanelli@ausl.re.it (M.Z.); 4 Second Department of Pathology, National and Kapodistrian University of Athens, Attikon University Hospital, Medical School, 12462 Athens, Greece; nkoufo@med.uoa.gr; 5 Gastroenterology and Endoscopy Unit, Azienda Ospedaliero Universitaria Policlinico “G. Rodolico—San Marco”, 95123 Catania, Italy; graziano.troina@libero.it (G.T.);

**Keywords:** eosinophilic esophagitis, chronic esophageal inflammation, endoscopic features, histopathology, therapeutic management

## Abstract

Eosinophilic esophagitis (EoE) has emerged as a distinct clinicopathological entity and a major cause of upper gastrointestinal morbidity worldwide. Once misinterpreted as a variant of Gastroesophageal Reflux Disease (GERD), its unique identity as an immune-mediated inflammatory disease was solidified by foundational studies in the early 1990s. This discursive review synthesizes key findings from peer-reviewed literature to outline the evolution of EoE’s diagnosis and management. The review highlights that a definitive diagnosis is now a multidisciplinary process that integrates clinical symptoms, characteristic endoscopic findings, and, most critically, a comprehensive histopathological evaluation of esophageal biopsies. It emphasizes the limitations of relying on a single eosinophil count and underscores the value of ancillary histological features and the Histologic Scoring System, which provides a more nuanced and predictive assessment of disease activity. The review also discusses the evolving understanding of PPI-responsive esophageal eosinophilia as a subtype of EoE, streamlining the diagnostic approach. In conclusion, while EoE is a chronic condition that can lead to significant esophageal remodeling if untreated, a robust diagnostic framework and a range of effective therapies are now available to manage the disease, though a continued lack of clinical awareness remains a key challenge.

## 1. Introduction

Eosinophilic esophagitis (EoE) has progressively emerged as a well-defined clinicopathological entity and is currently recognized as a major cause of upper gastrointestinal morbidity in both pediatric and adult populations [1]. Initially, the presence of eosinophils in the esophageal mucosa was considered a nonspecific histological finding and was historically attributed to gastroesophageal reflux disease (GERD) during the 1980s. Over the ensuing decades, however, substantial advances in clinical, pathological, and immunological research have led to the recognition of EoE as a distinct chronic, immune-mediated inflammatory disorder primarily confined to the esophagus [2].

The formal characterization of EoE as a separate disease entity dates back to the early 1990s, with seminal studies by Attwood et al. (1993) [3] in the United States and Straumann et al. (1994) [4] in Switzerland. These investigators independently described patient cohorts presenting with clinical manifestations and histopathological patterns distinct from those observed in GERD, thereby laying the foundation for EoE as a novel nosological category [3,4].

A critical turning point in understanding the etiopathogenesis of EoE was provided by Kelly et al. (1995), who reported a series of pediatric patients with GERD-like symptoms refractory to conventional medical and surgical interventions but who exhibited a striking clinical and histological remission following treatment with an elemental diet [5]. This pivotal observation established a direct link between food allergens and esophageal eosinophilic inflammation, thereby positioning EoE within the spectrum of antigen-driven, Th2-mediated allergic disorders.

**Figure 1:**
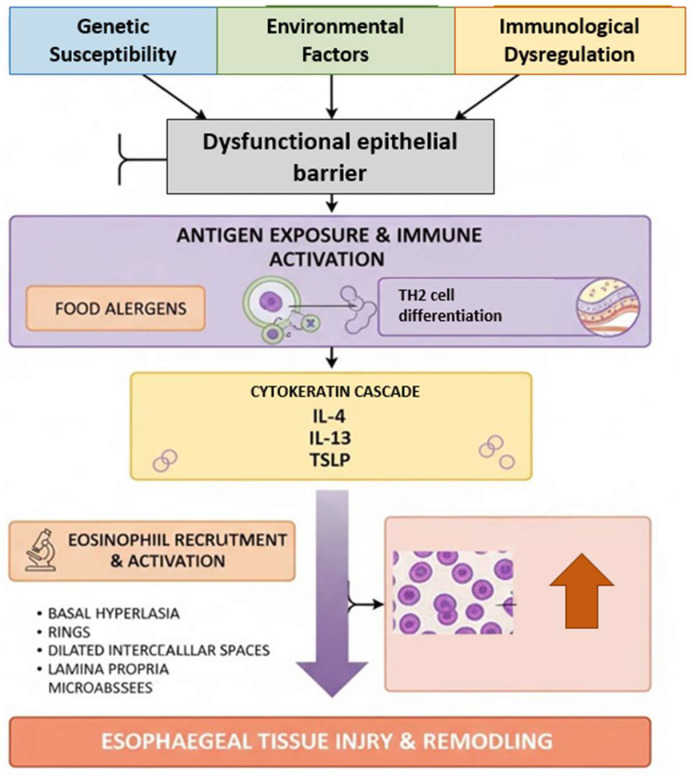
Pathogenesis of eosinophilic esophagitis.

EoE is now recognized as a chronic immune-mediated disease characterized by a complex interplay between genetic susceptibility, environmental triggers, and allergic sensitization. The esophageal inflammation is dominated by eosinophilic infiltration, leading to progressive tissue remodeling, subepithelial fibrosis, and, eventually, esophageal dysfunction. Clinically, this translates into dysphagia, food impaction, chest discomfort, and, in pediatric populations, feeding difficulties and failure to thrive. The chronicity of the inflammatory process often results in structural changes such as rings, strictures, and a narrow-caliber esophagus, which further exacerbate symptom severity and impair quality of life (Figure 1).

The growing body of clinical and translational research prompted the publication of the first international consensus guidelines on EoE in 2007, followed by an updated revision in 2011 to reflect new diagnostic criteria and therapeutic strategies [6,7]. Subsequently, major gastroenterological societies, including the American College of Gastroenterology (ACG, 2013) and the European and North American Societies for Pediatric Gastroenterology, Hepatology and Nutrition (ESPGHAN/NASPGHAN, 2014), released comprehensive evidence-based recommendations for both pediatric and adult patients [8]. These documents have standardized the diagnostic approach and highlighted the need for individualized management, incorporating dietary, pharmacological, and endoscopic interventions.

**Figure 2:**
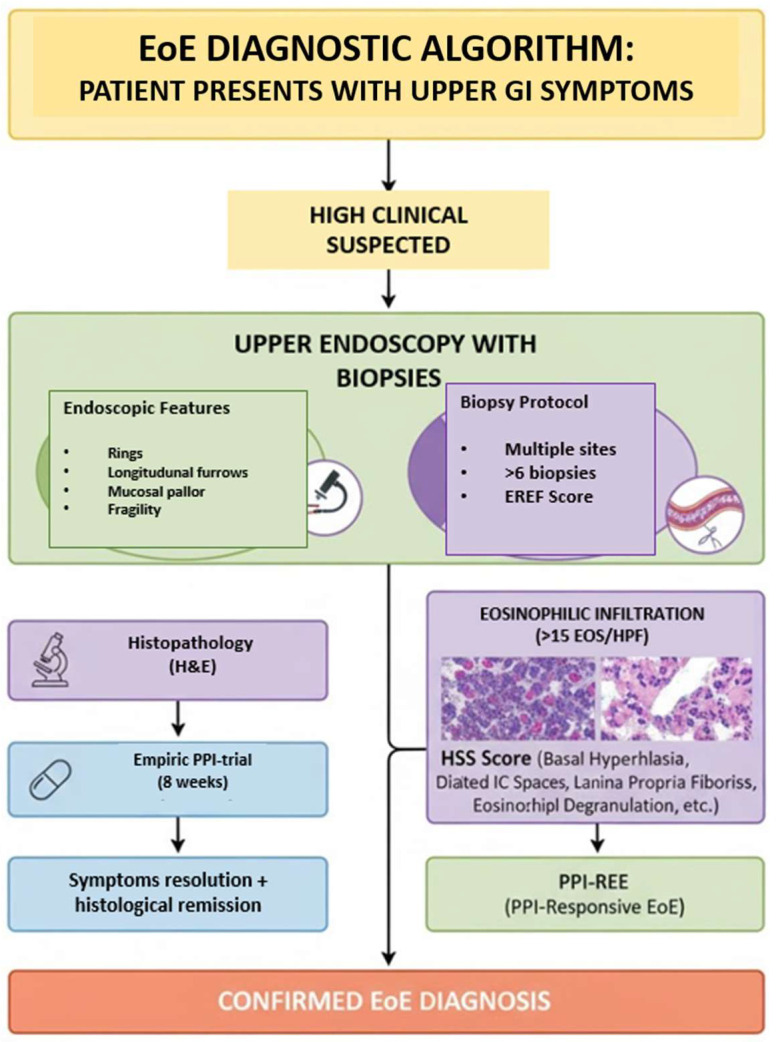
Diagnostic algorithm of eosinophilic esophagitis.

At present, the diagnosis of EoE relies on an integrated, multidisciplinary approach that combines clinical evaluation, endoscopic assessment, and histopathological confirmation. Endoscopic findings, such as linear furrows, white exudates, concentric rings, and mucosal fragility, provide important visual clues, but definitive diagnosis requires esophageal biopsies demonstrating a peak eosinophil count of ≥15 eosinophils per high-power field, in the absence of alternative causes of esophageal eosinophilia [9]. This diagnostic triad underscores the evolving understanding of EoE as a distinct, antigen-driven, esophageal inflammatory disease, whose recognition has profoundly reshaped the diagnostic and therapeutic landscape of upper gastrointestinal disorders (Figure 2).

### Natural History, Epidemiology, Diagnosis, Prognosis

The natural history of Eosinophilic Esophagitis (EoE) reveals a chronic, two-sided disease course. While many patients report a seemingly benign progression of symptoms (dysphagia) due to behavioral adaptations (like modified eating habits), objective data—specifically endoscopic and histological findings—show a more concerning reality. EoE is characterized by persistent inflammation that leads to slow but progressive fibrosis and stricture formation in the esophagus. Crucially, a longer duration of untreated disease is strongly associated with an increased risk and severity of these fibrostenotic complications. Therefore, long-term therapy is not just about symptom relief but primarily about preventing permanent structural damage by suppressing chronic inflammation [10].

In recent years, there has been an exponential increase in the incidence and prevalence of EoE, a phenomenon paralleled by a prolific rise in scientific literature. In the United States and Europe, EoE is now the most frequent cause of dysphagia in both children and young adults [11]. Prevalence varies considerably depending on the study population. However, this rises dramatically when the endoscopic procedure is performed specifically for symptoms like dysphagia or food impaction, reaching 63–88% in children and 10–15% in adults [12,13]. In the general population, the estimated prevalence is about 30–52 cases per 100,000 inhabitants [14]. While EoE has been described across all races and continents, a slight predominance is noted among Caucasians. The condition is also more frequent in males than females, with a typical ratio of 3:1, a difference that was even more pronounced (19:1) in one Spanish study [15]. The mean age at diagnosis typically ranges from 30 to 50 years in adults and 5.4 to 9.6 years in children. Interestingly, despite some proposed correlations with pollen exposure, a recent meta-analysis found no significant seasonal variation in the number of new diagnoses or episodes of food impaction [16].

The diagnosis of EoE hinges on a multidisciplinary approach (involving gastroenterology, allergology and immunology, pathology, dietetics, general surgery, and pediatric surgery) that integrates clinical symptoms, endoscopic observations, and, critically, the histopathological examination of esophageal biopsies. The presence of eosinophils in the esophagus is not an exclusive finding of EoE; therefore, a thorough differential diagnosis is essential to rule out other digestive and extra-digestive pathologies. The latest guidelines have refined this classification, no longer considering GERD and proton pump inhibitor-responsive (PPI-responsive) EoE as separate differential diagnoses [9]. This is because EoE and GERD can coexist, and PPI-responsive EoE is now recognized as a subtype of EoE, as it shares similar phenotypic and pathophysiological characteristics with the PPI-nonresponsive form. Consequently, a response to PPIs is no longer used as a differential diagnostic test but rather as a therapeutic option for EoE [17,18].

Treatment for clinically and histologically active EoE pursues two primary goals: first, to achieve symptom resolution to improve the patient’s quality of life; and second, to control inflammation to prevent progressive esophageal damage from tissue remodeling. In pediatric patients, a crucial additional objective is to ensure normal growth and development. Given its chronic nature, often compared to bronchial asthma, EoE necessitates a long-term therapeutic and follow-up strategy [17,18].

While eosinophilic esophagitis is not a life-threatening condition, if left untreated, it can cause permanent esophageal damage and significantly impair quality of life. The risk of developing esophageal stenosis increases with age and the delay in receiving a proper diagnosis and treatment. The most frequent complications include esophageal stenosis, food impaction, esophageal perforation, and malnutrition. There is currently no evidence to suggest that EoE is a premalignant condition or that it progresses to hypereosinophilic syndrome [19]. However, a recent association with esophageal granular cell tumors has been reported. The considerable delay often observed between symptom onset and diagnosis underscores a continued lack of clinical recognition, which has important implications for patient outcomes [20].

## 2. Materials and Methods

This discursive review was conducted through a comprehensive synthesis of the available peer-reviewed scientific literature pertaining to EoE, with particular emphasis on its histopathological features, diagnostic challenges, and evolving clinical paradigms. The objective was to integrate and critically interpret current evidence to delineate the key morphological, endoscopic, and clinical parameters that define this increasingly prevalent disease entity.

A systematic literature search was carried out using major biomedical databases, including PubMed, Scopus, and Web of Science, to ensure broad coverage of both clinical and translational research. The search strategy employed Medical Subject Headings (MeSH) and free-text terms such as “eosinophilic esophagitis”, “EoE”, “histopathology”, “diagnosis”, “biopsy”, “eosinophil count”, “endoscopy”, “differential diagnosis”, and “proton pump inhibitor-responsive esophageal eosinophilia”. Boolean operators (AND, OR) were used to optimize retrieval, and reference lists of relevant publications were hand-searched to identify additional pertinent studies. To ensure the timeliness and relevance of the data, the review primarily included articles published within the last ten years (2014–2024), encompassing original research studies, clinical trials, consensus statements, and expert guidelines issued by leading gastroenterological and allergological societies such as the American Gastroenterological Association (AGA), the American College of Gastroenterology (ACG), and the European Society for Paediatric Gastroenterology, Hepatology and Nutrition (ESPGHAN). Foundational or seminal studies predating this timeframe were selectively incorporated when they contributed substantially to the historical development, conceptual framework, or diagnostic standardization of EoE.

Inclusion criteria encompassed studies published in English-language peer-reviewed journals; research focusing on the diagnostic, histopathological, or endoscopic aspects of EoE; articles providing quantitative or qualitative data on eosinophil counts, biopsy techniques, or the interpretation of histological findings; and guideline documents and consensus statements offering standardized diagnostic algorithms.

Exclusion criteria included non-peer-reviewed publications, isolated case reports without histopathological confirmation, conference abstracts lacking full-text availability, and papers focusing solely on treatment without diagnostic relevance.

**Figure 3:**
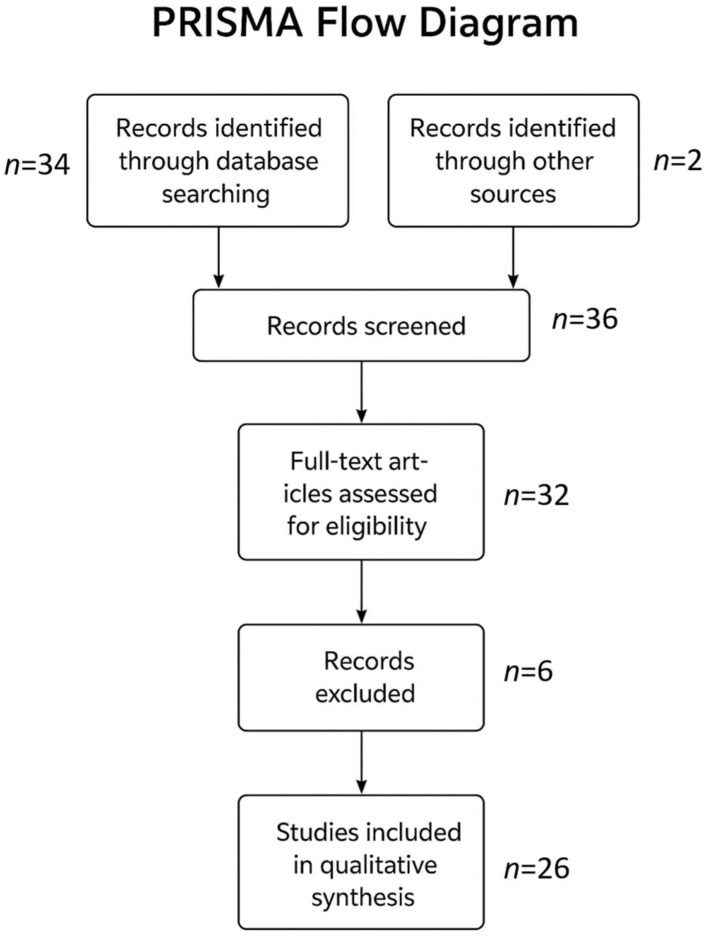
PRISMA flow diagram.

Each selected study was critically appraised for methodological quality, sample size, diagnostic criteria employed, and relevance to the aims of this review. Emphasis was placed on identifying recurring themes and knowledge gaps, particularly those relating to the variability in eosinophil distribution and its implications for biopsy sampling protocols; the diagnostic overlap with other causes of esophageal eosinophilia, including GERD and systemic eosinophilic syndromes; the evolving recognition of PPI-responsive esophageal eosinophilia as part of the EoE spectrum; and the application of standardized histologic scoring systems such as the HSS in disease evaluation and monitoring. Figure 3 shows how many studies were screened and, along with the criteria, which ones were included and excluded.

Data extracted from the literature were subsequently synthesized thematically to construct a coherent and integrative narrative. This approach allowed for the contextualization of findings across multiple disciplines—pathology, gastroenterology, immunology, and endoscopy—and enabled a comprehensive discussion of how histological insights translate into clinical practice. Given the inherently descriptive nature of this discursive review, no formal quantitative meta-analysis or statistical pooling of results was performed. Instead, emphasis was placed on qualitative synthesis, comparative interpretation, and critical reflection on the strengths, limitations, and emerging consensus within the existing body of evidence. The overarching goal was to provide a structured yet flexible framework that captures the current state of knowledge regarding the diagnostic and histopathological characterization of EoE while also highlighting areas where further research is warranted.

## 3. Results

**Figure 4:**
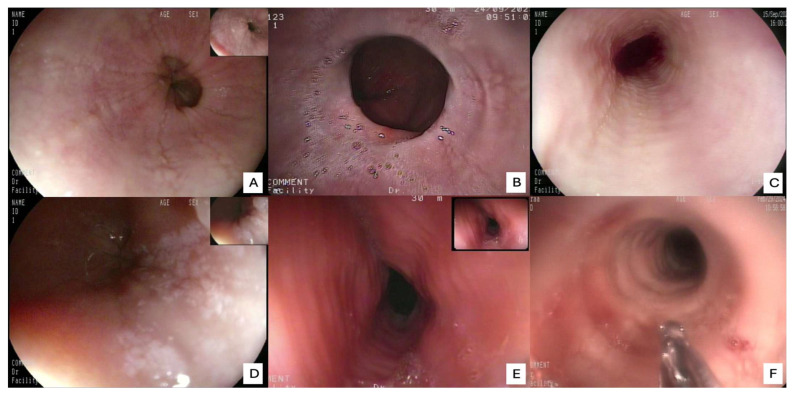
Typical endoscopic features of eosinophilic esophagitis. (**A**) Longitudinal furrows; (**B**) fibrotic stricture leading to luminal narrowing; (**C**) edema of the esophageal mucosa; (**D**) white exudates corresponding to eosinophilic microabscesses; (**E**,**F**) concentric rings (“fixed” or “transient” rings) giving the esophagus a corrugated appearance, which is also observed in cases of gastritis caused by nausea.

The review of the literature highlights several critical insights into the histopathology and diagnosis of EoE. The most consistent and defining histopathological feature is the presence of intraepithelial eosinophils, with a consensus threshold of ≥15 eosinophils/HPF in at least one esophageal biopsy [20]. However, it is widely recognized that eosinophil counts can be patchy, necessitating multiple biopsies from different esophageal levels (proximal, mid, distal) to enhance diagnostic yield. Beyond the eosinophil count, the presence of eosinophil microabscesses (clusters of ≥4 eosinophils) and the superficial layering of eosinophils are considered highly specific indicators of EoE. Other supportive histological findings frequently observed include basal zone hyperplasia (thickening of the basal layer, indicative of chronic inflammation), dilated intercellular spaces (spongiosis) reflecting epithelial edema, and lamina propria fibrosis, particularly in chronic cases, which correlates with esophageal remodeling and the development of strictures [21]. The presence and severity of these ancillary features, even at lower eosinophil counts, can aid in solidifying an EoE diagnosis when clinical and endoscopic suspicion is high. Endoscopic examination is crucial for visualizing the esophageal mucosa and guiding biopsy collection. While an endoscopically normal esophagus does not rule out EoE, several characteristic features are frequently observed: esophageal rings (trachealization), longitudinal furrows, white exudates or papules (representing eosinophil microabscesses), and strictures. The “crepe paper esophagus” (a fragile, easily torn mucosa) is also a significant endoscopic finding, especially in advanced cases (Figure 4). The combination of these macroscopic features with the appropriate histopathological findings forms the cornerstone of the diagnosis [22].

**Figure 5:**
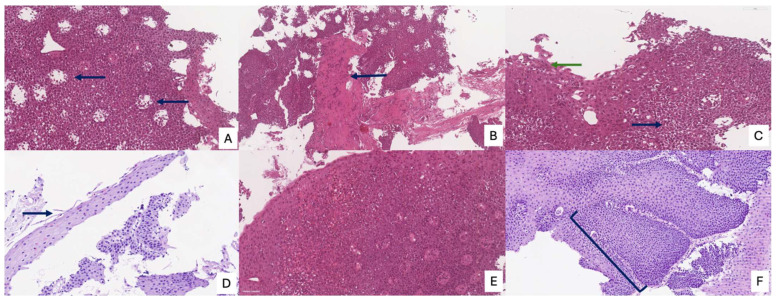
Histological and morphological features of eosinophilic esophagitis. (**A**) Eosinophilic abscesses (arrow); (**B**) lamina propria fibrosis (arrow); (**C**) superficial stratification of eosinophils (green arrow) and dilated intercellular spaces (blue arrow); (**D**) superficial epithelial alteration (arrow); (**E**) increased eosinophil density; (**F**) basal zone hyperplasia; (**A**–**F**) hematoxylin and eosin staining; original magnifications 50×.

**Figure 6:**
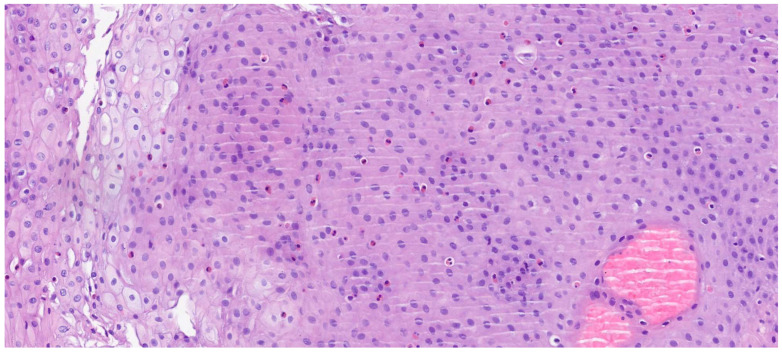
Esophageal mucosa with increased eosinophilia. Hematoxylin and eosin staining; original magnification 100×.

The Histologic Scoring System (HSS) for EoE represents a crucial and validated diagnostic and monitoring tool that provides a more nuanced and comprehensive assessment of the disease than a simple eosinophil count. This system is designed to evaluate eight specific histopathological criteria, each graded on a four-point scale from 0 (normal) to 3 (maximum variation), culminating in a total maximum score of 24. These criteria include eosinophil density, basal zone hyperplasia, the presence of eosinophil abscesses, superficial eosinophil stratification, dilated intercellular spaces, superficial epithelial alteration, dyskeratotic epithelial cells, and lamina propria fibrosis. Figure 5 and Figure 6 summarize the main histological features of eosinophilic esophagitis, according to the HSS score. By integrating these diverse pathological features, the HSS score effectively captures the full spectrum of tissue damage associated with EoE. Its predictive power is particularly significant, as it can identify relevant histological anomalies and monitor therapeutic response even in patients with low or absent eosinophil counts, a limitation of older diagnostic methods. Consequently, the HSS score offers a robust and accurate framework for evaluating disease severity and treatment efficacy, providing a more complete picture of the underlying pathological process [21]. Table 1 summarizes the principal histopathological aspects and criteria and their respective diagnostic importance.

A significant challenge in diagnosing EoE lies in differentiating it from other causes of esophageal eosinophilia, most notably GERD. While GERD can cause mild esophageal eosinophilia, it typically involves fewer eosinophils, a more diffuse distribution, and a prominent inflammatory infiltrate. The key diagnostic strategy to differentiate EoE from GERD involves a trial of high-dose PPIs for 8–12 weeks. Patients whose symptoms and esophageal eosinophilia resolve with PPIs are often classified as having PPI-responsive esophageal eosinophilia (PPI-REE). This entity is now widely considered part of the EoE spectrum, given shared clinical, endoscopic, and molecular features. Other conditions that can cause esophageal eosinophilia, though less commonly confused with EoE, include drug hypersensitivity reactions, hypereosinophilic syndrome, inflammatory bowel disease, and certain infections. A thorough clinical history, combined with careful endoscopic and histopathological evaluation, is essential to exclude these mimickers [17,18].

## 4. Discussion

The evolution in our understanding of EoE, from an obscure histological finding to a clearly delineated clinicopathological entity, has had profound and far-reaching implications for both clinical practice and patient outcomes. The reclassification of what was once regarded as “atypical GERD” into a distinct, immune-mediated inflammatory disorder has fundamentally reshaped diagnostic strategies, necessitating a more proactive, systematic, and multidisciplinary approach. This conceptual shift underscores the need for precision in both endoscopic and histopathological assessments to ensure timely diagnosis and appropriate therapeutic intervention. One of the major challenges consistently highlighted in the literature is the heterogeneous and patchy distribution of eosinophilic infiltration throughout the esophageal mucosa [9,22]. This histological variability makes reliance on a single biopsy both unreliable and potentially misleading, as it carries a substantial risk of false-negative results that may postpone diagnosis and delay effective management. Consequently, the current diagnostic standard of care mandates the acquisition of multiple biopsy specimens from different esophageal segments, typically from the proximal, mid, and distal regions, to maximize diagnostic yield and capture the full spectrum of mucosal involvement. This approach is not merely recommended but considered indispensable for accurate disease characterization.

**Figure 7:**
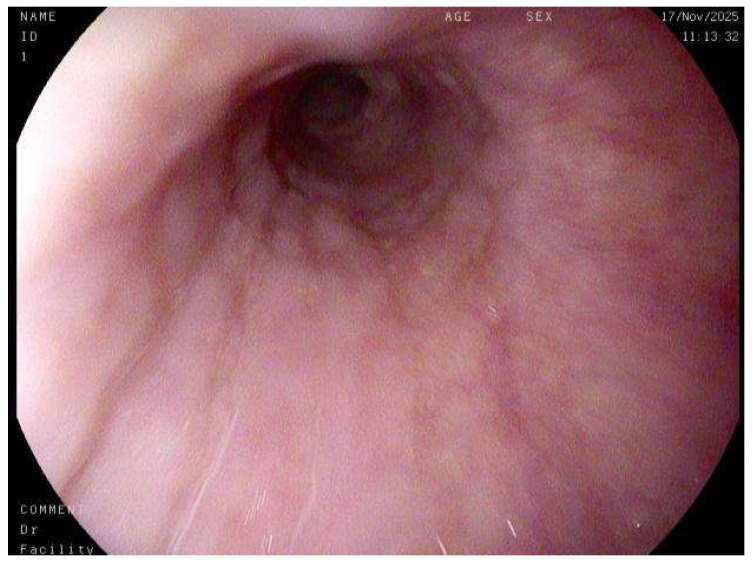
Endoscopy of a normal esophagus.

A nuanced interpretation of endoscopic findings has likewise become central to the diagnostic process. While a macroscopically normal-appearing esophagus (Figure 7) does not preclude the diagnosis of EoE, the identification of subtle yet characteristic endoscopic features, including esophageal rings (trachealization), longitudinal furrows, white exudates, mucosal pallor, and fragility, provides critical visual clues that can guide biopsy placement and enhance diagnostic accuracy [22]. It is therefore imperative that endoscopists maintain a high index of suspicion and possess adequate training to recognize these early morphological indicators, even in the absence of overt fibrostenotic remodeling or stricture formation. The incorporation of standardized endoscopic scoring systems, such as the EoE Endoscopic Reference Score (EREFS), has further contributed to diagnostic uniformity and interobserver reliability, facilitating more objective disease evaluation in both clinical and research settings.

The development of the HSS represents a pivotal advancement, moving the diagnostic paradigm beyond the simplistic reliance on peak eosinophil counts toward a multidimensional, semi-quantitative assessment of tissue injury [21]. The HSS encompasses eight distinct histopathological domains, including features such as basal zone hyperplasia, dilated intercellular spaces, eosinophil degranulation, and lamina propria fibrosis, each weighted according to severity. This system allows for a more holistic and reproducible evaluation of disease activity, providing clinicians with a valuable tool to monitor both inflammatory and remodeling components over time. Notably, the presence of lamina propria fibrosis has been shown to correlate directly with the duration and intensity of chronic inflammation and represents a key predictor of fibrostenotic complications, including esophageal strictures, a principal determinant of long-term morbidity in EoE [21]. By integrating these ancillary histopathological features, the HSS enhances both diagnostic precision and the capacity to quantitatively assess therapeutic response, paralleling the structured disease-monitoring frameworks used in other chronic allergic and inflammatory conditions, such as asthma.

Another major conceptual advance has been the evolving understanding of PPI-REE as part of the EoE disease spectrum rather than as a separate entity [17,18]. This reclassification has simplified both the diagnostic and therapeutic algorithms, positioning PPIs as a legitimate and effective first-line treatment for many patients. PPIs not only suppress gastric acid secretion but also exert anti-inflammatory effects, including modulation of epithelial barrier function and attenuation of Th2 cytokine signaling. This dual mechanism of action underscores the drug’s role as both a diagnostic aid and a therapeutic intervention, reinforcing its early use in the EoE treatment continuum.

Despite these advances, a persistent challenge remains the significant diagnostic delay often observed between the onset of symptoms and the establishment of a definitive diagnosis [19]. Numerous studies have documented median delays of several years, during which ongoing inflammation may lead to irreversible tissue remodeling and esophageal fibrosis, thereby complicating management and reducing treatment efficacy. This gap in recognition reflects not only the variable clinical presentation of EoE but also a continued lack of awareness among healthcare professionals across multiple specialties.

Therefore, sustained educational efforts and cross-disciplinary collaboration are imperative to improve early detection and intervention. Increasing awareness through targeted training programs, consensus-based diagnostic algorithms, and guideline dissemination will be vital in bridging the diagnostic gap, optimizing therapeutic outcomes, and ultimately improving the long-term quality of life for individuals affected by this chronic and increasingly prevalent disease.

## 5. Conclusions

In conclusion, EoE is now firmly established as a distinct, chronic, and potentially progressive immune-mediated disorder of the esophagus that demands a comprehensive and multidisciplinary approach for both diagnosis and long-term management. Over the past three decades, the understanding of EoE has evolved remarkably, transitioning from an underrecognized histological finding to a well-characterized clinical entity with defined pathophysiological mechanisms. This evolution has been driven by an exponential increase in scientific literature, fostering the development of robust diagnostic frameworks and evidence-based management strategies.

The contemporary diagnostic paradigm for EoE integrates clinical symptomatology, endoscopic features, and, most importantly, histopathological assessment, which remains the cornerstone of diagnosis. Recognition of subtle histological parameters, such as basal zone hyperplasia, lamina propria fibrosis, and eosinophilic microabscesses, together with the use of standardized scoring systems such as the HSS, has greatly enhanced diagnostic accuracy and facilitated the stratification of disease severity [17,18,21]. This multidisciplinary framework underscores the complexity of EoE, where immunologic, genetic, and environmental factors converge to drive a chronic inflammatory cascade confined to the esophageal mucosa.

Although EoE is not a life-threatening condition, its chronic, relapsing nature can lead to irreversible esophageal remodeling, resulting in fibrostenotic complications such as strictures and a narrow-caliber esophagus, which significantly impair swallowing function and quality of life [23]. Consequently, timely diagnosis and sustained disease control are essential to prevent progression toward structural damage. However, delayed clinical recognition remains a persistent challenge, with many patients experiencing prolonged diagnostic latency, often spanning several years between symptom onset and definitive diagnosis [23]. This delay underscores the need for enhanced awareness among primary care physicians, gastroenterologists, allergists, and pathologists to ensure earlier identification and intervention.

**Figure 8:**
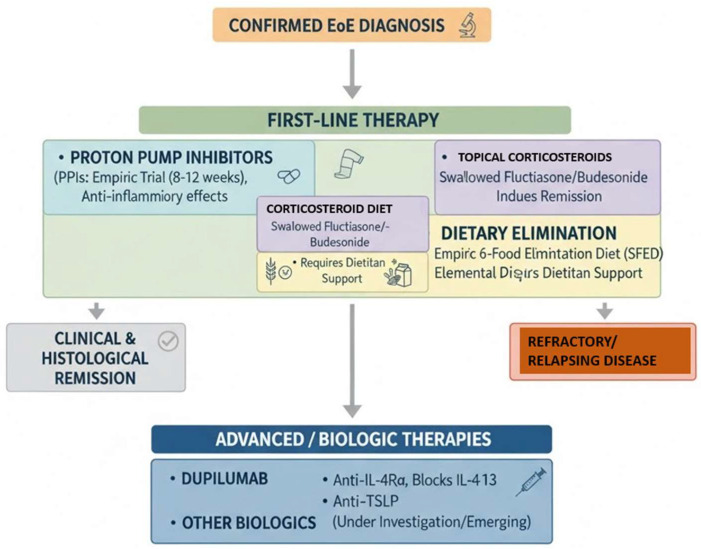
Latest treatment algorithm for eosinophilic esophagitis.

From a therapeutic standpoint, the current armamentarium encompasses a wide spectrum of effective interventions, ranging from dietary elimination therapies (including elemental and empiric six-food elimination diets) to topical corticosteroids and proton pump inhibitors, all of which have demonstrated efficacy in inducing histological and clinical remission. More recently, the advent of biologic agents, particularly monoclonal antibodies targeting key cytokine pathways such as IL-4 and IL-13 (e.g., dupilumab), has opened new horizons for patients with refractory or relapsing disease, offering sustained remission with favorable safety profiles [22]. Nevertheless, the optimal sequencing and duration of therapy, particularly in cases of disease recurrence or partial response, remain areas of ongoing investigation and debate (Figure 8).

### Future Directions

Future research efforts should aim to further elucidate the molecular and immunological underpinnings of EoE, identifying novel biomarkers capable of predicting disease activity, therapeutic response, and risk of progression. Advances in genomic, transcriptomic, and proteomic profiling hold promise for the development of precision medicine approaches that could tailor therapy to individual patient phenotypes and endotypes. Additionally, the integration of noninvasive diagnostic tools, such as minimally invasive esophageal sampling devices, serum biomarkers, and imaging-based assessments, may reduce the reliance on repeated endoscopic biopsies, improving patient compliance and long-term monitoring.

Equally important, future studies should focus on long-term outcome data to define optimal treatment duration, evaluate relapse prevention strategies, and assess the real-world effectiveness of emerging biologic therapies [24,25]. Ultimately, sustained collaboration among clinicians, pathologists, and basic scientists will be crucial to translate these insights into clinical practice, advancing toward a more personalized and preventive model of care for patients with eosinophilic esophagitis [26].

## Figures and Tables

**Table 1:** Key diagnostic histopathological features of EoE.

Histopathological Feature	Criterion	Diagnostic Value
Intraepithelial Eosinophils	≥15 eosinophils per HPF in at least one esophageal biopsy.	Cardinal diagnostic criterion. While necessary, it is not sufficient alone. Counts can vary; multiple biopsies (proximal, mid, distal) recommended due to patchy distribution. Lower counts may be significant if other features are present.
Eosinophil Microabscesses	Clusters of ≥4 eosinophils within the intraepithelial space.	Highly specific for EoE. Often found in the superficial layers of the epithelium. Indicates significant localized eosinophilic inflammation.
Superficial Layering of Eosinophils	Eosinophils preferentially accumulate in the superficial epithelial layers.	Characteristic spatial distribution, distinguishing EoE from other eosinophilic infiltrations, where eosinophils might be more diffusely spread or concentrated deeper.
Basal Zone Hyperplasia	Thickening of the basal layer of the squamous epithelium to >20% of total epithelial thickness.	Represents increased epithelial turnover and chronic inflammation. Common in EoE but can also be seen in GERD; thus, it is a supportive feature, not diagnostic on its own.
Dilated Intercellular Spaces	Widening of spaces between epithelial cells (spongiosis).	Reflects epithelial edema and breakdown of barrier function. Contributes to esophageal dysfunction and is a sign of acute epithelial injury.
Lamina Propria Fibrosis	Increased collagen deposition in the lamina propria.	Correlates with chronic, untreated disease and esophageal remodeling (e.g., strictures). Indicates irreversible structural changes, contributing to mechanical complications.
Subepithelial Fibrosis	Fibrosis present beneath the epithelial layer.	Less common but can be a subtle indicator of chronic inflammation and remodeling.
Loss of Epithelial Integrity	Erosion, ulceration, or desquamation (less common in routine biopsies due to fragility).	While not a primary diagnostic feature, it reflects severe inflammation and can be related to food impaction or instrumentation.

## Data Availability

No new data were created or analyzed in this study. Data sharing is not applicable to this article.

## Abbreviations

The following abbreviations are used in this manuscript:

EOE	Eosinophilic esophagitis
GERD	Gastroesophageal Reflux Disease
HSS	Histologic Scoring System
MeSH	Medical Subject Headings
PPI	Proton pump inhibitor
PPI-REE	Proton pump inhibitor-responsive esophageal eosinophilia
EREFS	Endoscopic Reference Score
AGA	American Gastroenterological Association
ACG	The American College of Gastroenterology
ESPGHAN	The European Society for Paediatric Gastroenterology, Hepatology and Nutrition
